# A low dimensional embedding of brain dynamics enhances diagnostic accuracy and behavioral prediction in stroke

**DOI:** 10.1038/s41598-023-42533-z

**Published:** 2023-09-21

**Authors:** Sebastian Idesis, Michele Allegra, Jakub Vohryzek, Yonatan Sanz Perl, Joshua Faskowitz, Olaf Sporns, Maurizio Corbetta, Gustavo Deco

**Affiliations:** 1 Center for Brain and Cognition (CBC), Department of Information Technologies and Communications (DTIC), Pompeu Fabra University, Edifici Mercè Rodoreda, Carrer Trias i Fargas 25-27, 08005 Barcelona, Catalonia Spain; 2 Padova Neuroscience Center (PNC), University of Padova, via Orus 2/B, 35129 Padua, Italy; 3 Department of Physics and Astronomy “G. Galilei”, University of Padova, via Marzolo 8, 35131 Padua, Italy; 4 Department of Neuroscience, University of Padova, via Giustiniani 5, 35128 Padua, Italy; 5 Veneto Institute of Molecular Medicine (VIMM), via Orus 2/B, 35129 Padua, Italy; 6 Department of Psychological and Brain Sciences, Indiana University, Bloomington, IN 47405 USA; 7 Universidad de San Andrés, Buenos Aires, Argentina; 8 National Scientific and Technical Research Council, Buenos Aires, Argentina; 9 Institut du Cerveau et de la Moelle Épinière, ICM, Paris, France; 10 Centre for Eudaimonia and Human Flourishing, Linacre College, University of Oxford, Oxford, UK

**Keywords:** Cognitive neuroscience, Computational neuroscience, Computational models, Neurological disorders

## Abstract

Large-scale brain networks reveal structural connections as well as functional synchronization between distinct regions of the brain. The latter, referred to as functional connectivity (FC), can be derived from neuroimaging techniques such as functional magnetic resonance imaging (fMRI). FC studies have shown that brain networks are severely disrupted by stroke. However, since FC data are usually large and high-dimensional, extracting clinically useful information from this vast amount of data is still a great challenge, and our understanding of the functional consequences of stroke remains limited. Here, we propose a dimensionality reduction approach to simplify the analysis of this complex neural data. By using autoencoders, we find a low-dimensional representation encoding the fMRI data which preserves the typical FC anomalies known to be present in stroke patients. By employing the latent representations emerging from the autoencoders, we enhanced patients’ diagnostics and severity classification. Furthermore, we showed how low-dimensional representation increased the accuracy of recovery prediction.

## Introduction

There is growing evidence that neural activity is low-dimensional, at various scales, in agreement with theoretical work arguing that low-dimensional dynamics exist in the brain because neural circuits operate more effectively in low dimensions^1,2^. This suggests that the huge dimensionality of functional neuroimaging data (tens of thousands of voxels or hundreds of regions) may be highly redundant, and that it may be possible to find low-dimensional representations, or “latent signals”^3^ preserving most of the relevant information content. However, evidence that a low-dimensional representation can actually retain the most prominent dynamical features, including, crucially, clinically relevant features, is still weak.

In this work, we use dimensionality reduction, which maps high-dimensional data into a new space whose dimensionality is much smaller^4^, to investigate low-dimensional representations of fMRI data in normal subjects and stroke patients. This is an ideal testbed to assess the clinical relevance of low-dimensional representation. At the population level there is strong evidence that post-stroke neurological impairments have a low-dimensional structure^5^. Moreover, stroke produces dysfunction in distributed brain networks^6^, which are commonly identified as low-dimensional abnormalities in functional connectivity (FC) that predict behavioral deficits after stroke. These include reductions in interhemispheric network integration, ipsilesional network segregation, and network modularity^7–14^.

Classic dimension reduction techniques, such as PCA, are linear. When applying a linear method to neural activity data and keeping “d” dimensions, one is implicitly assuming that the neural activity sits on a “d”-dimensional plane. However, the actual shape—the manifold—on which the neural activity sits is generally a curved surface, and nonlinear^15^. Machine learning can be employed to find low-dimensional, nonlinear representations of complex data with only a small set of latent variables. While several previous studies have applied deep learning in fMRI data^16–18^, they have mainly focused on classification accuracy, rather than low-dimensional representations. Here, we apply deep learning methods directly to functional time series to extract a nonlinear low-dimensional representation of brain dynamics. In addition, we leverage the recently proposed Temporal Evolution NETwork (TENET) framework^19,20^ to analyze the asymmetry, or “reversibility” in the flow of the brain signals. TENET offers critical insight about the degree of non-equilibrium in brain dynamics, a variable that has been shown to be severely altered in consciousness disorders^20^ and could be of high clinical significance in other neurological conditions. TENET requires to compare cross-correlation matrices, whose estimation is affected by large error in high dimensional spaces, and therefore is expected to significantly profit from a dimensionality reduction step.

Here, we will demonstrate that the latent non-linear components of brain dynamics found by machine-learning approaches retain the most important dynamical features that are usually identified from the high-dimensional original data. Furthermore, we will demonstrate that the latent representation is more powerful, both for diagnostic and for prognostic purposes. Diagnostically, the latent representation yields a better classification of clinical status (healthy/mild—stroke/severe stroke) at the acute stage, and a better prediction of behavioral deficit. Prognostically, it improves the prediction of recovery after 1 year of the incident, as compared to other methods.

## Materials and methods

### Subjects

We used the Washington University Stroke Cohort dataset^21^, a large prospective longitudinal (two weeks, three months, 12 months) study of first-time single lesion stroke in different locations. The database includes patients with first-time stroke, studied at 1–2 weeks (mean = 13.4 days, SD = 4.8 days), 3 months, and 12 months after stroke onset. Furthermore, a group of age-matched control subjects was evaluated twice at an interval of three months. From this cohort we selected 96 stroke patients and 27 healthy subjects.

Stroke patients were prospectively recruited from the stroke service at Barnes-Jewish Hospital (BJH), with the help of the Washington University Cognitive Rehabilitation Research Group (CRRG). The complete data collection protocol is described in full detail in a previous publication^21^. Healthy control group was typically constituted of spouses or first-degree relatives of the patients, age- and education-matched to the stroke sample. Patients were characterized with a robust neuroimaging battery for structural and functional features, and an extensive (~ 2 h) neuropsychological battery.

The participants were selected based on the same inclusion/exclusion criteria as in^21^ briefly mentioned below:

Inclusion Criteria. (1) Age 18 or greater. No upper age limit. (2) First symptomatic stroke, ischemic or hemorrhagic. (3) Up to two lacunes, clinically silent, less than 15 mm in size on CT scan. (4) Clinical evidence of motor, language, attention, visual, or memory deficits based on neurological examination. (5) Time of enrollment: < 2 weeks from stroke onset. (6) Awake, alert, and capable of participating in research.

Exclusion criteria. (1) Previous stroke based on clinical imaging. (2) Multi-focal strokes. (3) Inability to maintain wakefulness in the course of testing. (4) Presence of other neurological, psychiatric, or medical conditions that preclude active participation in research and/or may alter the interpretation of the behavioral/imaging studies (e.g., dementia, schizophrenia), or limit life expectancy to less than 1 year (e.g., cancer or congestive heart failure class IV). (5) Report of claustrophobia or metal object in body.

### Neuroimaging acquisition and preprocessing

We use data from the Washington University Stroke Cohort, extensively described in previous articles^13,21–24^. A brief description of the data acquisition and preprocessing follows. A complete description of it is explained in detail in a previous publication^14^.

Neuroimaging data were collected at the Washington University School of Medicine using a Siemens 3T Tim-Trio scanner with a 12-channel head coil. It was obtained sagittal T1-weighted MP-RAGE (TR = 1950 ms; TE = 2.26 ms, flip angle = 90 degrees; voxel dimensions = 1.0 × 1.0 × 1.0 mm), and gradient echo EPI (TR = 2000 ms; TE = 2 ms; 32 contiguous slices; 4 × 4 mm in-plane resolution) resting-state functional MRI scans from each subject. Participants were instructed to fixate on a small centrally located white fixation cross that was presented against a black background on a screen at the back of the magnet bore. Seven resting-state scans (128 volumes each) were obtained from each participant (~ 30 min total) giving a total of 896 time points for each participant.

Resting-state fMRI preprocessing included (i) regression of head motion, signal from ventricles and CSF, signal from white matter, global signal, (ii) temporal filtering retaining frequencies in 0.009–0.08 Hz band. This frequency band is a standard for rs-fMRI studies and is the default band used in the most widespread software studies and connectivity, the CONN toolbox^25^; and (iii) frame censoring, FD = 0.5 mm. Finally, the resulting time series were projected on the cortical and subcortical surface of each subject divided into 235 ROIs (200 cortical plus 35 subcortical). The atlas and the functional labels for each ROI can be obtained together with the scripts used in the current manuscript at: https://github.com/SebastianIdesis/Latent_Space_Stroke-2023-.

These areas are taken from the multi-resolution functional connectivity-based cortical parcellations developed by Schaefer and colleagues^26^, including additional subcortical and cerebellar parcels from the Automated Anatomical Labeling (AAL) atlas^27^ and a brainstem parcel from the Harvard–Oxford Subcortical atlas (https://fsl.fmrib.ox.ac.uk/fsl/fslwiki/Atlases).

A structural connectome atlas was created using a publicly available diffusion MRI streamline tractography atlas based on high angular resolution diffusion MRI data collected from 842 healthy Human Connectome Project participants^28^ as described previously^11^.

### Stroke deficit assessment

#### Lesion volume and severity

Lesion volume was calculated based on the topography of stroke using a voxel-wise analysis of structural lesions. Each lesion was manually segmented on structural MRI scans and checked by two board certified neurologists. The location (cortico-subcortical, subcortical, white-matter) of each individual lesion was assigned with an unsupervised K-means clustering on the percentage of total cortical/subcortical gray and white matter masks overlay. The overlap of each lesion group with gray matter, white matter and subcortical nuclei is explained in detail in a previous publication (see^21^). Furthermore, the structural disconnection information consisted of a sparse connection adjacency matrix where each cell quantified the percentage of streamlines connecting each region pair in the atlas-based structural connectome that were unharmed by the lesion. Therefore, the multiplication of each structural disconnection matrix with a template SC provides an atlas-based weight for each region pair corresponding to each patient^24^.

In addition to the anatomical lesion volume, the patients’ clinical severity was assessed through the National Institutes of Health Stroke Scale (NIHSS)^29^ that includes 15 subtests addressing: level of consciousness (LOC), gaze and visual field deficits, facial palsy, upper and lower motor deficits, limb ataxia, sensory impairment, inattention, dysarthria and language deficits. The total NIHSS score was used as an averaged measure of the clinical severity for each patient. The lesion volume relation with the NIHSS was assessed, showing a significant association. In addition, we inspected the total disconnection tracks relation with lesion volume, which also revealed a significant relation (Supp. Fig. 1). The metric of total disconnection tracks was described in detail in previous literature^14^.

#### FC abnormalities

Local ischemia, which damages cells and neural connections at the site of injury, primarily affects white matter, thus altering long-range FC between cortical areas. Three types of large-scale FC alterations affect Resting State Networks (RSNs)^13^: (i) within-network interhemispheric FC^9,11,13,14^, (ii) between-network intra-hemispheric FC^7,8,10,13^; and (iii) Modularity^10,12,22^.

### Autoencoder

An autoencoder takes an input with a high dimensionality, processes it through a neural network and tries to compress the data into a smaller representation^18^. In order to achieve this, the procedure takes two steps: encoder (embedding) and decoder (reconstruction). The autoencoder, therefore, consists of a deep neural network with rectified linear units as activation functions and dense layers, which bottlenecks into the d-dimensional layer^30^. Gradient descent was implemented to backpropagate the errors, with the purpose of training the network. The minimized loss function consists of a canonical reconstruction error term (calculated from the output layer of the decoder).

Subsequently, to acquire training and test sets, we produced 80/20% random splits. We employed the training set to optimize the autoencoder parameters. The training process involved batches with 256 samples and 100 training epochs (if needed) making use of a loss function and an Adam optimizer^30^.

**Figure 1:**
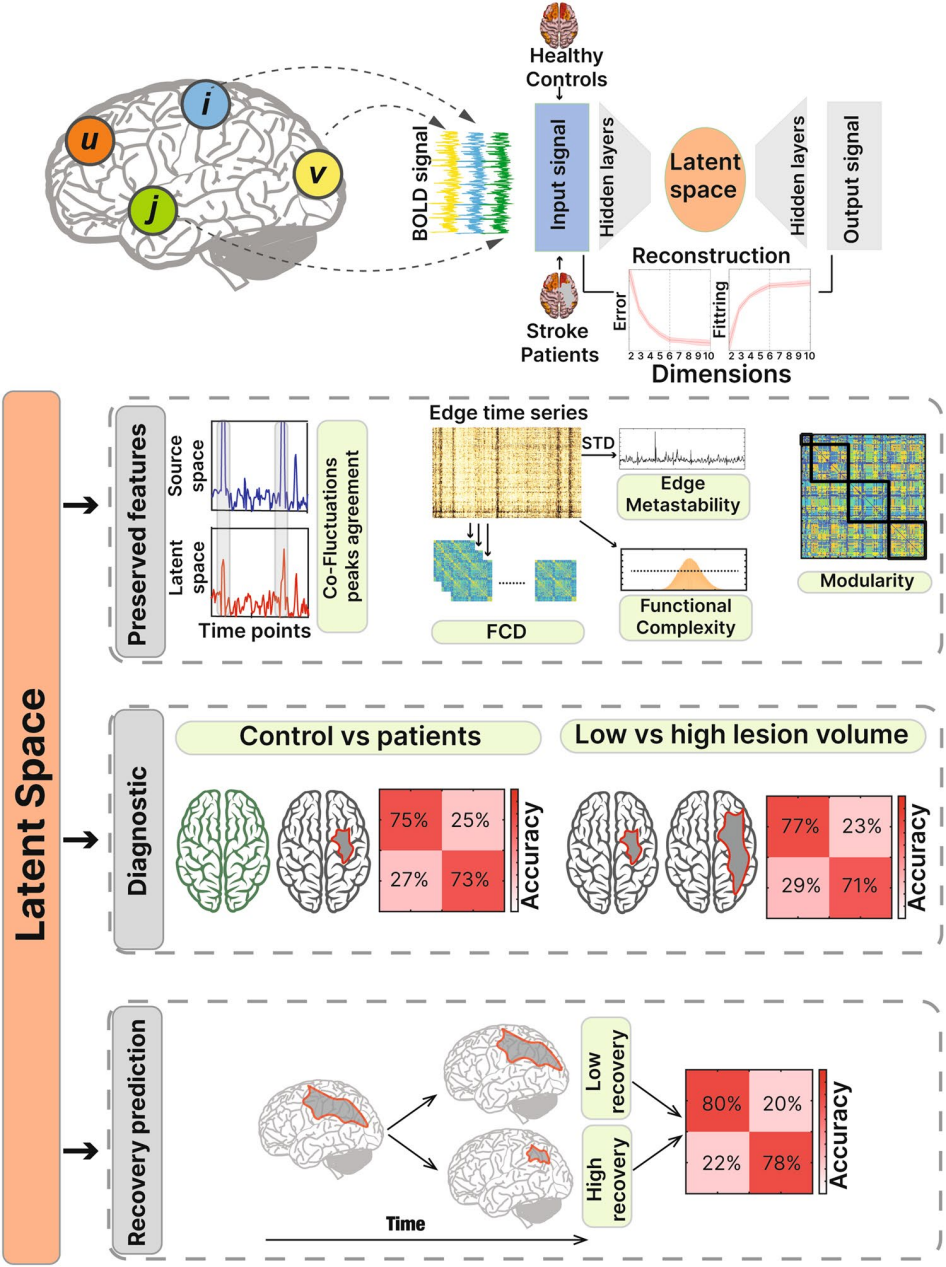
Summary of the analysis. fMRI signals from both groups (healthy controls and stroke patients) were used as input signals for the autoencoder. Reconstruction error was calculated by assessing the difference between the output and the input signal. The latent information was used to perform three different analyses: Top segment shows the features both in source and latent space to verify if the information was preserved after the dimensionality reduction. Middle segment shows the accuracy of the classification used to separate healthy controls from stroke patients and to separate stroke patients with low and high lesion volume. Lower segment shows how the latent representation is used to predict the recovery after one year after the stroke.

The encoder network applies a nonlinear transformation to map the input signal into Gaussian probability distributions in latent space, and the decoder network mirrors the encoder architecture to produce reconstructed matrices from samples of these distributions. By observing the reconstruction loss (comparison between the output and input signal), the performance of the autoencoder could be tuned to the appropriate hyperparameter configuration (Supp. Fig. 2a). Autoencoders have proven to be effective even when applied on small sample sizes^31^, such as the one considered here (time points x subjects = 48,384). In order to avoid overfitting, we applied 10 iterations of cross-validation (80/20) and early stopping techniques (as the performance of the model increases to a peak point, training can be stopped^32^) As input for the autoencoder we used the BOLD signal of both healthy controls and stroke patients (Fig. 1a). Therefore, the input (and the output) of the autoencoder consists of a matrix with the number of ROIs as the number of rows and the concatenated time points of the subjects’ time series as columns (every subject provides 896 samples for the AE training). This results in a 235-by-896 matrix of data representing each subject. Nevertheless, for the classification and prediction analyses, different metrics were used as input (see section “Classification”).

### Edge-centric analysis

Using a straightforward unwrapping of the Pearson correlation, co-fluctuation time series (alternatively referred to as “edge time series”) data can be estimated for each edge. Unlike sliding-window time-varying connectivity, which requires the parameterization of a window duration, kernel shape, and step size, edge time series have the same temporal resolution as the original time series data. Importantly, the time-averaged value of edge time series is the correlation coefficient. This means that edge time series are a mathematically exact decomposition of a functional connection into its framewise contributions. Previous analyses of edge time series data have shown that transient periods of high-amplitude activity make disproportionately large contributions to the time-averaged functional connectivity^33–36^. In other words, data selected from specific temporal slices can be used to reconstruct a similarity matrix with a high correspondence to the functional connectivity matrix constructed from the full dataset^37,38^.

In the current study, we applied a peak detection algorithm as previously implemented^39^. The collective co-fluctuations of brain regions were estimated as the root sum square (RSS) of co-fluctuations between all pairs of brain regions (edges) at every given time point. Next, BOLD time series were randomly shifted (using MATLAB’s circshift operator), hence approximately preserving each node’s autocorrelation, while randomizing the cross-correlation across nodes. This null model was iterated 1000 times. Time points in the original time series for which the empirically observed RSS amplitude exceeded the null model (P < 0.001) were maintained. The resulting peaks in the original RSS at the corresponding time points were considered as significant events. RSS peaks that exceed extreme z-score values (above or below 4.5 deviations) were excluded from the analysis. These peaks do not occur frequently (at most once per 1100 frames). The correlation between the FC created by the timepoints containing peaks, and the original FC, gives an indication of how much information is contained in these specific points^23^.

Edge-centric analysis has been applied to stroke datasets in a previous study^23^. This work demonstrated that edge-centric measures, such as normalized entropy or high-amplitude co-fluctuations (transient periods of high-amplitude activity), can be used as indicators of lesion severity and recovery^23^.

### Dynamic features preserved/enhanced in latent space

#### FCD

Time versus-time matrix representing the functional connectivity dynamics (FCD), where each entry FCD(t1, t2) is defined by a measure of resemblance between FC(t1) and FC(t2)^40,41^. Therefore, the FCD captures the spatiotemporal organization of FC by representing the coincidences between FC(t) matrices. It results in a symmetric matrix where an entry (ts1, ts2) is defined by the Pearson correlation between FC(ts1) and FC(ts2)^42^.

#### Edge metastability

We calculated the standard deviation of the edge time series which represents the temporal metastability. This metric gives information about temporal variability in the level of synchronization^43,44^.

#### Modularity

Overall Newman’s modularity was calculated, making a comparison between the number of connections within a module to the number of connections between modules^45^. We adopted a constant null derived from the Potts model^46^. We retained the full FC matrix, including its negative entries, for the purpose of community detection by applying the Louvain algorithm. Louvain bipartitions are identified by first inspecting a wide range of the resolution parameter, selecting upper and lower boundaries within which a two-community structure occurs, followed by a finer sampling of the range to retrieve bipartitions.

#### Functional complexity

Functional complexity was calculated based on previous literature^47^. According to this definition of the feature, complexity emerges when the collective dynamics are characterized by intermediate states, between independence and global synchrony^48^. Thus, the authors choose to define complexity as the difference between the observed distribution of the functional connectivity and a uniform distribution. Hence, functional complexity is quantified as the integral between the two distributions. The latter is estimated by approximating the distributions with histograms and replacing the integral with the sum of differences over the bins. The equation for the functional complexity is given below; for more information, see^47^.

1 $$C = 1 - \frac{1}{{c_{m} }} \mathop \sum \limits_{\mu = 1}^{m} \left| {p_{\mu } (r_{ij} ) - \frac{1}{m}} \right| ,$$

where |.| means the absolute value and $C_{m } = 2 \frac{m - 1}{m}$ is a normalization factor that represents the extreme cases in which the p($r_{ij}$) is a Dirac-delta function $\delta_{m}$.

### Reversibility

Reversibility was computed by assessing the difference between the time-shifted correlation matrices for the forward and reversed time series, which reflects the level of non-equilibrium. In previous publications, significantly lower levels of reversibility were found in deep sleep and anaesthesia compared to wakefulness^20^. This approach provides a quantification of the level of nonreversibility and consequently the degree of non-equilibrium in the brain dynamics of different brain states, or in the case of this study, different groups (controls vs stroke patients).

Particularly, the assumed causal dependency between the time series *x*(*t*) and *y*(*t*) is measured through the time-shifted correlation. For the forward evolution the time-shifted correlation is given by

2 $$C_{forward} \;(\Delta t) = \left\langle {x(t),\;y(t + \Delta t)} \right\rangle$$

And for the reversed backward evolution the time-shifted correlation is given by

3 $$C_{reversal} \;(\Delta t) = \langle {x^{(r)} (t),\;y^{r} (t + \Delta t)} \rangle$$

being $\Delta t = 1$ based on previous literature^20^. This amounts to the minimum step size (one frame for the window) within which irreversibility is calculated taking away the arbitrary selection of a time window duration.

Where the reversed backward version of *x*(*t*) (or* y*(*t*)), that we call $x^{(r)} (t)$ (or $y^{(r)} (t)$), is obtained by flipping the time ordering.

The pairwise level of non-reversibility is given consequently by the absolute difference between the assumed causal relationship between these two timeseries in the forward and reversed backward evolution, at a given shift $\Delta t = 1$. For the current study the shifting was selected at T = 1.

4 $$I_{x,y} \;(T) = |C_{forward} \;(T) - C_{reversal} \;(T)|$$

Therefore, the level of irreversibility relies on the idea of finding the arrow of time through the degree of asymmetry obtained by comparing the lagged correlation between pairwise time series.

The extent to which the forward and reversed time series are distinguishable determines the reversibility/equilibrium level. Thus, when the forward and reversed time series are not distinguishable, the system is reversible and in equilibrium, whereas when the level of distinguishability increases, the system becomes more irreversible and away from the equilibrium.

In summary, the selected approach (Temporal Evolution NETwork—TENET) captures the non-equilibrium of brain activity quantified by the non-reversibility of the signal (difference between the “forward” and “reversed” signal). For more information, see^19^.

### Classification

A random forest classifier^30,49^ was used in order to classify the participants. Briefly, the random forest algorithm builds upon the concept of a decision tree classifier, where samples are iteratively split into two branches depending on the values of their features^30,49^.

For the classification procedure, there were two different division criteria:

1. Distinction between controls and stroke patients.
2. Distinction between patients with high vs low lesion volume (see section: “Stroke deficit assessment”). As an alternative, we calculated the distinction using thee NIHSS as division criteria, showing similar results (Supp. Fig. 10).

We trained random forest classifiers with 1000 decision trees using 80% of the subjects through cross-validation analysis. All accuracies were determined as the area under the receiver operating characteristic curve (AUC).

**Figure 2:**
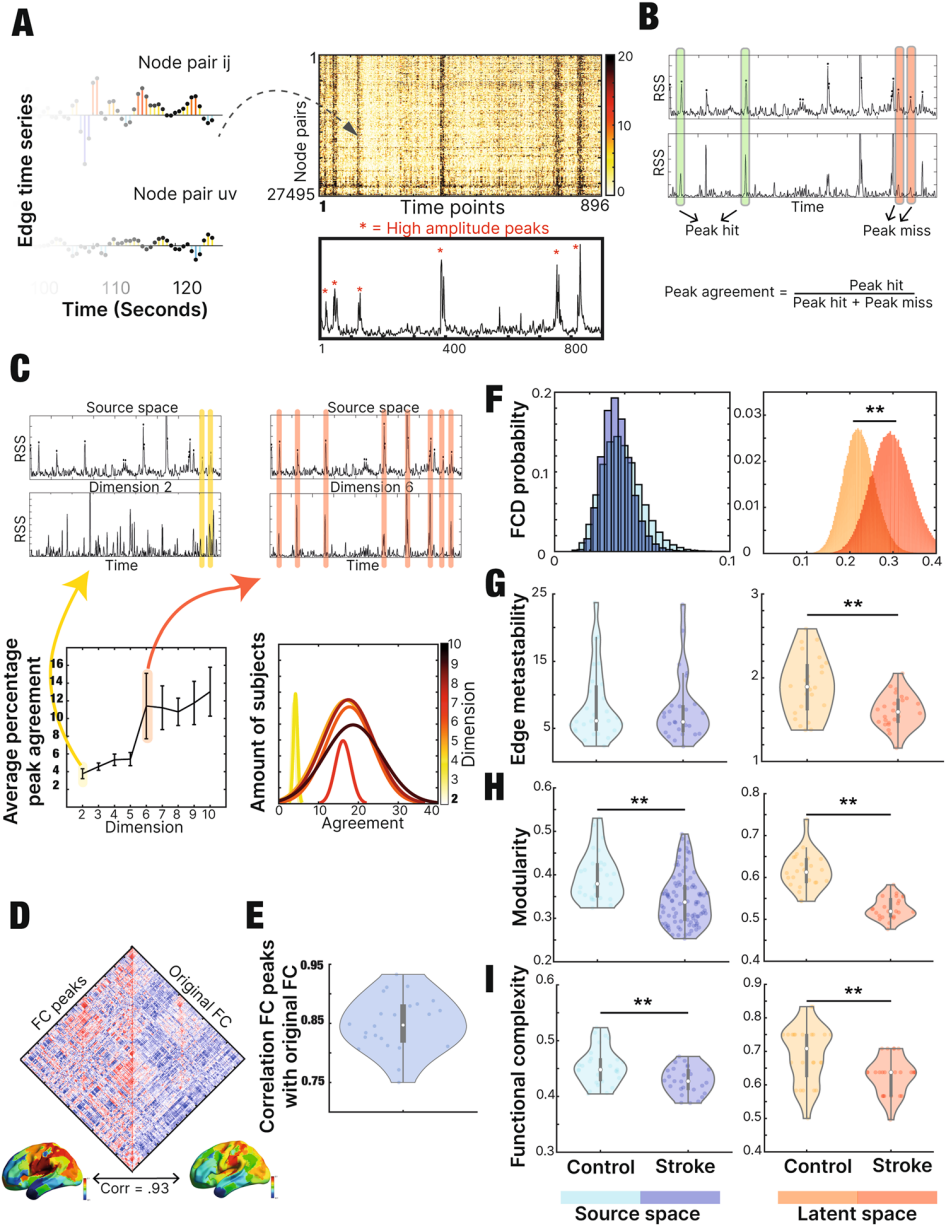
Preserved features in latent space: (**A**) Calculation of edge time series by means of Hadamard product at each time point. The highest top amplitude frames (top %10 of co-fluctuation root-sum-squared) were selected. (**B**) The collective co-fluctuations of brain regions were estimated as the root sum square (RSS) of co-fluctuations between all pairs of brain regions (edges) at every given time point. The peaks that occur at the same time point in the source and latent space were labeled as “peak hits” and used to calculate the “peak agreement”. This amount was used as an indicator of preserved dynamics. (**C**) The amount of peak agreement was calculated for each dimension revealing dimension 2 as the lowest. There was a significant difference between dimension 5 and 6 (*p* < 0.01). Following dimension 6, the level of agreement stabilizes. (**D**) The original FC was contrasted with the FC created only by the timepoints containing peaks, showing a correlation of R = 0.93. The FC strength (average of each node) was projected onto surface in order to observe the spatial pattern similarity. (**E**) The correlation between the FC of the timepoints containing peaks and the complete FC was performed for all the subjects in order to characterize the variability across subjects. (**F**) We compared the distribution of the FCD of healthy controls and stroke patients. The difference observed in source space (*p* < 0.01) increased when observing the latent representation (*p* < 0.01). (**G**) Edge metastability was compared between controls and stroke patients showing no significant difference in the source space (*p* = 0.9) in contrast to the latent space (*p* < 0.01). (**H**) Level of modularity was significantly different in controls and stroke patients both in source (*p* < 0.01) and latent space (*p* < 0.01). (**I**) Level of functional complexity was significantly different in controls and stroke patients both in source (*p* < 0.01) and latent space (*p* < 0.01).

The same procedure was applied both in source and latent space, having as possible features either the upper triangle of the corresponding FC, or the upper triangle of the reversibility matrix (see “Reversibility” section). The latent space information used was obtained from the latent dimension 6 due to the results obtained in Fig. 2c and Supp. Fig. 2.

The reversibility matrix is calculated as defined in previous literature^19,20^ in which the difference is calculated between the signal in “real arrow of time” compared to the “reversed arrow of time”. The resulting measure captures how different, or “asymmetrical”, the signal is across time. Key demographic variables such as age and gender may strongly affect our findings. Therefore, we verified that the two groups (patients/controls) were matched in terms of these variables (Supp. Table 1). In addition, to further control for the possible confounding influence of these factors, we checked that the two metrics showing a significant patients/control difference did not present any significant age or gender effect. To this aim, we divided subjects by age (using the corresponding median) and gender. In both cases, we found no significant differences (p > 0.2) between the groups (Supp. Fig. 13).

### Latent space visualization through 2D-projection

**Figure 3:**
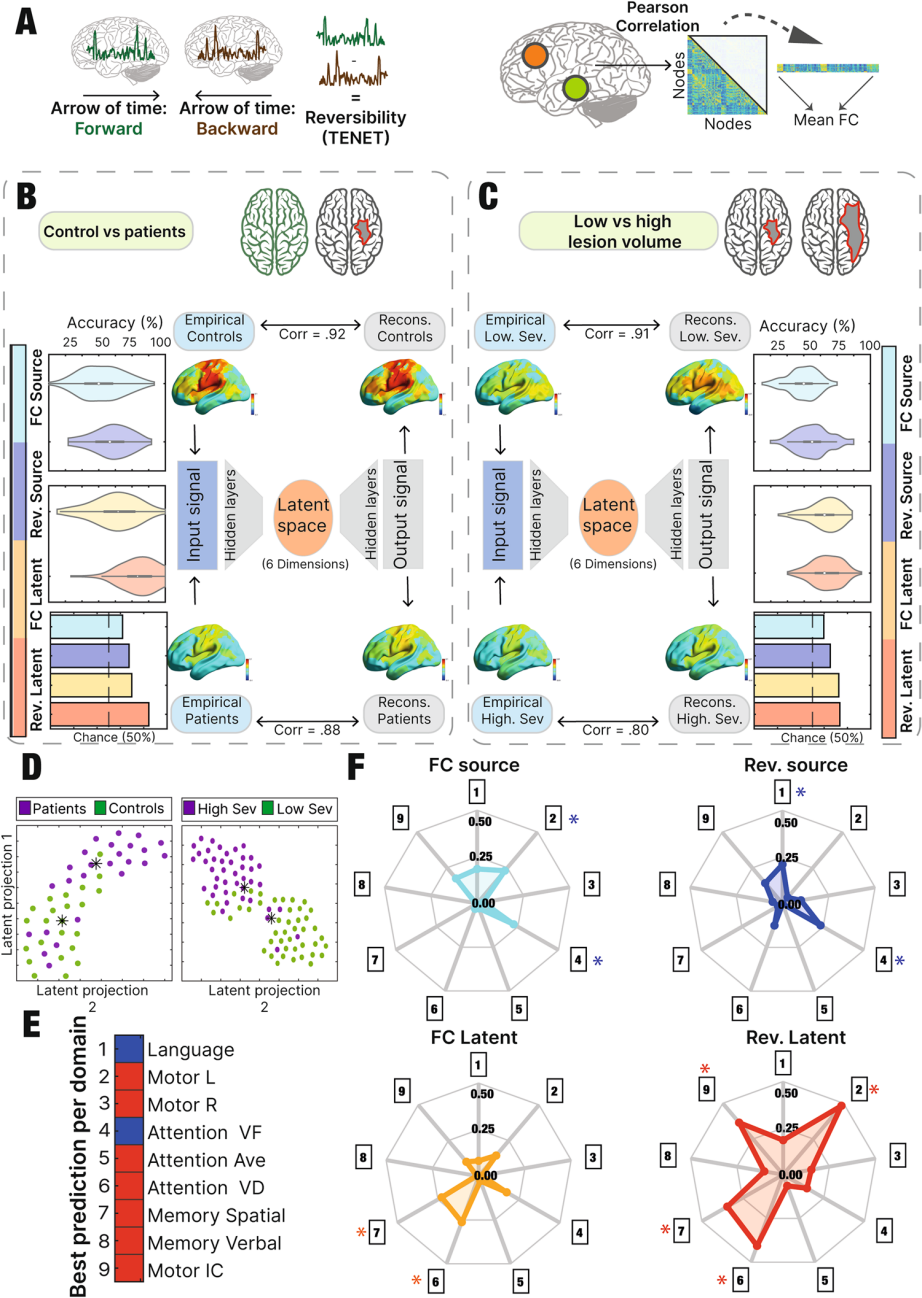
Classification in acute stage and relation with behavior: (**A**) Reversibility was computed by calculating the average of the difference between the time-shifted correlation matrices for the forward and reversed time series. Mean FC was calculated from the mean of the upper triangle of the FC. (**B**) The classification between controls and patients at the acute stage showed the reversibility in the source space as the highest accuracy (mean = 79%). The right part of the panel shows the comparison between the input and the output signal of the autoencoder. (**C**) The distinction between stroke patients with low and high lesion volume indicated that the highest accuracy was given by the reversibility in the latent space (mean = 73%). Same description of the autoencoder as in panel (**B**) was presented in panel (**C**). (**D**) 2-dimension projection of the latent representation obtained in the controls’ vs patients’ latent space (left) and the patients with low vs high lesion volume latent space (right). Asterisks represent the mean of each group. (**E**) All the metrics used for the classification approach were related with each of the 9 behavioral domains recovery values (score after 1 year minus score after 2 weeks, divided the 2 weeks score). Asterisks represent which of the relations were significant. (**F**) For each behavioral domain, the corresponding metric with the highest association was represented indicating the respective color. Red represents the latent space metric while blue represents the source space.

In order to visualize the distribution of the subjects and obtain a more intuitive understanding of their variability, we converted the data in the latent space into a two-dimensional plane. In order to achieve this goal, we used t-Distributed Statistic Neighbor Embedding (t-SNE)^50,51^. The proposed approach aims to preserve the local and global data structure while visualizing all samples in a two-dimensional plane. The higher-dimensional data is transformed into a set of pairwise similarities and embedded in two dimensions such that similar samples are grouped together^51^. Figure 3d shows this approach performed both in the separation of controls versus patients, and patients with high versus low severity of damage. It is relevant to clarify the different amount of participants in both figures, as the first scatterplot is a comparison between a subset of patients (to have the same amount of subjects per group) against the healthy controls (the dataset consists of 27 healthy controls) while the second is a comparison within all the patients presented in the dataset (96 patients).

### Neuropsychological and behavioral assessment

The same subjects (controls and patients) underwent a battery of neuropsychological tests in the domains of motor, attention, language, visual, and memory functions at each time point. Briefly, the battery consisted of 44 measures across four domains of function: language, motor attention and memory (for a complete description of the tasks measures, see^21^). A dimensionality reduction was applied to the individual test data in each domain using principal component analysis as in^21^, yielding summary domain scores: Language, MotorR and MotorL (one score per side of the body), AttentionVF (visuospatial field bias), Average performance (overall performance and reaction times on attention tasks), and AttentionValDis (the ability to re-orient attention to unattended stimuli), Memory V (composite verbal memory score) and MemoryS (composite spatial memory score). Finally, patients’ behavioral scores were z-scored with regards to controls’ scores, to highlight behavioral impairments.

In addition to domain-specific scores, the patients’ clinical severity was assessed through the National Institutes of Health Stroke Scale (NIHSS)^29^ that includes 15 subtests addressing: level of consciousness (LOC), gaze and visual field deficits, facial palsy, upper and lower motor deficits, limb ataxia, sensory impairment, inattention, dysarthria and language deficits. The total NIHSS score was used as an averaged measure of the clinical severity for each patient.

### Association of reduced dimensions with each behavioral domain

The relation between each of 9 behavioral tasks and the features used for the subjects’ classification (see “Classification” section) was assessed by means of Pearson correlation.

The presented result in Fig. 3f was performed with 6 dimensions. Furthermore, Supp. Fig. 3 shows the same analysis with 10 dimensions in order to demonstrate the influence of higher dimensions when using dimensionality reduction approaches.

### FC distance

In order to measure the similarity or distance between the stroke patients FC (at each time point) and the healthy controls FC, we used the Frobenius norm of the difference between the two FC matrices. The higher the FC Distance, the higher the damaging impact of lesion on FC. Similarly, the lower the FC Distance, the lower is the impact^52^. As an alternative approach to calculate the FC, we used the pairwise co-classification of nodes for a consensus clustering procedure (Supp. Fig. 4). Co-classification yields a full matrix of pairwise affinities between nodes (scaled between 0 and 1) and one can thus use existing community detection algorithms for the consensus clustering step^53^. This analysis was added as a control as it has proven to overcome existing problems while identifying community structures^53^.

### Correlation FC/SC

For each subject, the structural–functional coupling metric was quantified using Pearson’s correlation between structural (healthy control anatomical template, see “Methods”: “Subjects”) and functional connection strengths as reported in previous studies^54–56^.

Previous work has shown how the acute stages after stroke incidents reveal a low relation between the two matrices while the strength of their relation increase as the patients recovered which enable its use as a metric of recovery across time^54^. As reported in previous studies^57^, we calculated the correlation between the SC and FC values for each network in order to assess how the stroke incident altered the structural–functional coupling in the corresponding areas (Supp. Fig. 5).

### Prediction of recovery

A random forest classifier was used in order to classify the participants based on their recovery, comparing the ones with high level (better recovery) against low level (worse recovery). For more details, see section “Classification”. The same procedure was applied both in source and latent space, using as possible features either the upper triangle of the corresponding FC, or the upper triangle of the reversibility matrix. The latent space information used was obtained from latent dimension 6 due to the results obtained in Fig. 2c.

The division between high vs low recovered was made by splitting the sample in two using the median of three distinct metrics.

1. Behavior: Amounts of domains recovered after 1 year.
2. FC Distance: Distance between the subjects FC after 1 year and the healthy control FC.
3. Correlation FC-SC: FC-SC correlation of each subject after 1 year.

As an alternative for the behavioral metric, we applied a principal component analysis of the behavioral recovery scores ((1 year–2 weeks)/2 weeks) as used in previous literature^58^. We used the first component’s median as a separation criterion (Supp. Fig. 6).

### Relationship of each dimension with FC abnormalities

We estimated the relation between previously explained anatomical and functional features (see section “Stroke deficit assessment”) with the mean and standard deviation of each time-pattern in the latent space giving as a result 30 possible associations (5 features × 6 dimensions). Results are presented in Supp. Fig. 7.

### Latent FC means per dimension

The Pearson correlation was calculated at the latent space with all the estimated dimensions giving as an output a matrix of D × D (Dimensions). The mean of the resulting matrix was obtained in order to compare across the different dimensions of the latent space. (Supp. Fig. 2b).

## Results

We derived a low-dimensional embedding of the BOLD signal of a group of healthy controls and stroke patients and analyzed the latent information contained in it (Fig. 1). The technique calculates the reconstruction error at each latent space dimension (2 through 10) to identify the point at which the error stabilizes (Supp. Fig. 2a). The correlation between the source and the latent space exceeds the value of 0.9 at dimension 6 (see section “Preserved features in latent space”). Once obtained the latent representation, the resulting matrix of size D × T (Dimensions by time points) of the testing set is used to analyze the embedded properties of the signal. As a first step, several functional features were calculated in both source and latent space to determine whether the information is preserved after the dimensionality reduction. Next, the latent representation was used to classify healthy controls vs patients at the acute stage (2 weeks after the stroke incident). Last, the low-dimensional embedding at the acute stage was used to predict recovery, in order to compare the recovery prediction accuracy with that obtained with the source space signal. The present study shows how the embedded information obtained through autoencoders can improve well-studied metrics of diagnostic and prediction of recovery.

### Preserved features in latent space

The latent representation obtained through the autoencoder exposed how non-trivial dynamic features were preserved. To illustrate this point, we selected 5 different metrics that demonstrate various time series attributes (Co-fluctuations peak agreement, Functional Connectivity Dynamics, Edge metastability, Modularity, and Functional complexity). In all cases, the features were maintained, and in some cases, enhanced. For some of the analysis, we focus on a specific dimensionality, with 6 dimensions, based on the results provided by the reconstruction error calculation (Supp. Fig. 2a). It is worth clarifying that as the latent reduction requires a division between training and testing dataset, the amount of subjects used for the latent space is slightly lower, as visible in the different degrees of freedom. The same analyses were performed using a classical linear dimensionality reduction method, principal component analysis (PCA). To compare PCA with the latent representation obtained through the autoencoder, we used the same number of dimensions for the two methods, considering the 6 first principal components. These components accounted for only 85 percent of the total variance, implying that the autoencoder achieved a more efficient degree of dimensional compression. The results showed a higher performance of the autoencoder compared to PCA in preserving the dynamical features and discriminating between healthy controls and stroke patients (Supp Fig. 14). This suggests that the non-linear dimensionality reduction achieved though the autoencoder is much more efficient at capturing relevant dynamical features.

In order to assess the amount of temporal information that was preserved after the dimensionality reduction, we first calculated the edge time series (see “Methods” section) (Fig. 2a). Furthermore, we compared how many time points classified as peaks coincided in both spaces (peaks agreement). The metric was normalized by dividing it by the sum of Peak Hits (coincidence) plus Peak Miss (not coincidence) (Fig. 2b). We calculated the metric across all latent dimensions. There is a significant increase in the peak agreement between dimension 5 and dimension 6 (*t*(170) = − 8.75, *p* < 0.01), while no other consequent dimension exhibited significant differences revealing how the level of agreement stabilized after reaching dimension 6 (Fig. 2c). Remarkably, the stabilization of the peak agreement coincided with the one observed in the reconstruction error. These findings support the selection of 6 as the optimal dimension for performing the following analysis.

We compared the co-fluctuation high-amplitude peaks (see “Methods” section: “Edge-centric analysis”) in source and latent space. It has been reported that the aforementioned peaks contain a large amount of the signal information^36,39^. A way to assess this is by observing the correlation between the FC component, created by the timepoints containing peaks, and the original FC. Therefore, we calculated the association between the standard FC and the FC computed only using the timepoints that revealed to contain peaks, resulting in a maximum Pearson correlation value of R = 0.93 (Fig. 2d). Furthermore, the same correlation was displayed for all subjects showing an average association of R = 0.84 (Fig. 2e).

We compared the distribution of FCD between controls and stroke patients, revealing a significant difference in the source space (*t*(1790) = − 7.89, *p* < 0.01). Nevertheless, the difference gets enhanced when comparing in the latent representation (*t*(1790) = − 36.20, *p* < 0.01)) (Fig. 2f).

While there is no significant difference in the source space while comparing the level of edge metastability between controls and stroke patients (*t*(52) = 0.12, *p* = 0.9), the difference is significant when comparing in the latent representation (*t*(46) = 3.5, *p* < 0.01) (Fig. 2g).

While there is a significant difference in the source space when comparing the level of Modularity between controls and stroke patients (*t*(121) = 4.48, *p* < 0.01), the difference is enhanced when comparing Modularity in the latent representation (*t*(46) = 8.77, *p* < 0.01)) (Fig. 2h).

While there is a significant difference in the source space when comparing the level of Functional Complexity between controls and stroke patients (*t*(52) = 3.23, *p* < 0.01), the difference is comparable when calculating Functional Complexity in the latent representation (*t*(46) = 4.1, *p* < 0.01) (Fig. 2i).

In summary, the latent representation highlights crucial dynamical differences between patients and controls.

### Classification in acute stage and relation with behavior

We assessed patients’ diagnostics and severity classification through machine learning algorithms. We performed the same analysis in both source, and latent space, in order to compare if the embedded information in the latent representation was informative. To achieve that goal, we relied on two different metrics, the widely used FC mean, and the brain signal reversibility (see “Methods”) as a novel metric inferring signal dynamics and complexity. The results revealed how the dimensionality reduction benefits both the metrics, but specially the reversibility one, which obtained the maximum classification accuracy. Furthermore, the content of the latent representation was projected into 2-dimensions (see “Methods”) in order to visualize how the group separation is better described in a non-linear way (Fig. 3d).

We used a random forest classifier to discriminate healthy controls from stroke patients at the acute stage. As an input to the classifier, we considered two summary metrics, the reversibility (assessing the degree of reversibility of BOLD time series, see “Methods”), and the mean FC, comparing the results in the source and latent space. The latent information used for this analysis was explained in detail in the “Methods” section. As an alternative, we performed the same analysis but replacing the mean FC by the standard deviation, showing similar results (Supp. Fig. 11).

The mean FC and the reversibility matrix were selected as metrics as they integrate spatial and temporal dynamics, together with the complexity of the system. Furthermore, this study aimed to use a traditional metric (mean FC) next to a novel one (reversibility), which has already been used with promising results in previous publications^19,20^.

Reversibility in the latent space showed the highest accuracy performance (mean = 0.84, SD = 0.11), followed by Reversibility in the source space (mean = 0.70, SD = 0.10), mean FC in the latent space (mean = 0.67, SD = 0.13) and mean FC in the source space (mean = 0.61, SD = 0.11) (Fig. 3b).

While classifying between stroke patients with high and low lesion volume of damage, reversibility in the latent space showed the highest accuracy performance (mean = 0.73, SD = 0.09), followed by mean FC in the latent space (mean = 0.72, SD = 0.09), Reversibility in the source space (mean = 0.65, SD = 0.10) and mean FC in the source space (mean = 0.59, SD = 0.09) (Fig. 3c).

We searched for the association between the previously reported metrics and all the behavioral scores differences normalized ($\frac{1{\text{ year score}}-2{\text{ weeks score}}}{2{\text{ weeks score}}}$) assessing the degree of behavioral recovery (Fig. 3f). Furthermore, we performed the same analysis but using the behavioral scores at the acute stage (2 weeks) in order to see the relations at the initial state (Supp. Fig. 8). Each radar plot shows the relation of each metric with each of the 9 behavioral domains. The further the point is from the center, the higher the Pearson correlation value is. Furthermore, significant relations are indicated with asterisks (Fig. 3f).

When using the FC at the source space, there was a significant relation with Motor L (*r* = 0.25, *p* < 0.05) and Attention VF (visuospatial field bias, referred as “AttVF”) (*r* = 0.24, *p* < 0.05). When using the Reversibility at the source space, the following domains showed a significant relation: Language (*r* = − 0.21, *p* < 0.05), and AttentionVF (*r* = 0.23, *p* < 0.05). When using the FC at the latent space, the following domains showed a significant relation: AttValDis (the ability to re-orient attention to unattended stimuli) (*r* = − 0.26, *p* < 0.05) and memory S (spatial memory) (*r* = − 0.22, *p* < 0.05). When using the reversibility at the latent space, the following domains showed a significant relation: MotorL (*r* = 0.48, *p* < 0.05), AttValDis (*r* = 0.40, *p* < 0.05), MemoryS (*r* = 0.34, *p* < 0.05) and Motor IC (*r* = 0.36, *p* < 0.05) (Fig. 3f). Behavioral tasks abbreviations are explained in detail in the “Methods” section.

In summary, the reversibility matrix got the highest number of significant relations with behavioral domains (4), followed by the reversibility matrix in source space, the FC matrix in latent space and the FC matrix in source space with 2 significant relations. We performed the same analysis but replacing the average FC for the standard deviation showing a higher value in the relationships’ strength (Supp Fig. 9). Mean FC and standard deviation are global metrics that allow the comparison of the source space and the latent representation, converting them in ideal approaches to assess the comparison between the two spaces.

Lastly, each domain’s highest association is visualized in Fig. 3e, displaying the highest Pearson correlation value for each behavioral variable. As a control, we inspected the same analysis while replacing the chosen dimension. The result of the association between dimension 10 and the 9 behavioral scores is presented in Supp. Fig. 3.

### Prediction of recovery

We intended to assess recovery of stroke patients across one year in 3 different ways.

We aimed to study the patients’ behavior, next to their functional dynamics and their functional-structural coupling. As in the previous analysis, we used FC mean and reversibility of the signal as distinctive metrics, and we performed the calculations in both source and latent space in order to demonstrate how latent information predicts better the recovery of the patients, especially when using the reversibility as metric. As an alternative, we performed the same analysis but replacing the mean FC with the standard deviation, showing similar results (Supp. Fig. 12).

**Figure 4:**
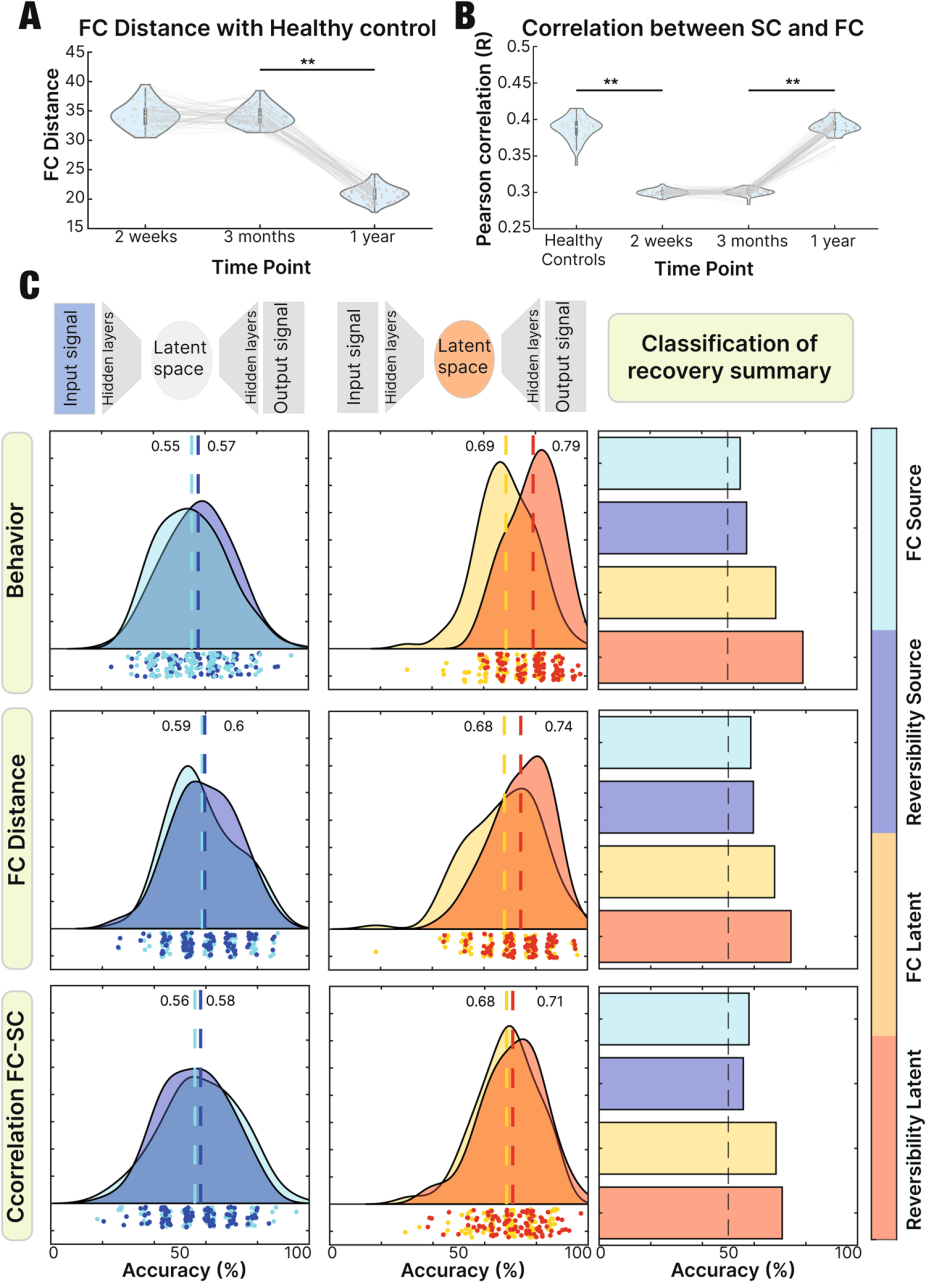
Prediction of recovery: (**A**) FC distance (Frobenius norm of the difference between the two matrices) between stroke patients at each time point and the healthy controls indicating the decrease of distance after 1 year (*p* < 0.01). (**B**) Correlation between SC and FC of healthy controls and stroke patients (at each measurement stage) revealing the increase at the remote stage, showing a similar value to controls after 1 year of the incident (*p* < 0.01), while it is not the case after 2 weeks and 3 months (*p* = 0.86). (**C**) Prediction of recovery using as input of the classifier the FC and reversibility matrix of the source space and the FC and reversibility matrix of the latent space. To split the subjects in high vs low recovered, 3 different criteria were used: Amount of behavioral domains recovered, the FC distance at remote stage and the correlation between SC-FC at remote stage. In all the scenarios, reversibility in the latent space showed the highest accuracy.

To assess the recovery using functional information, for each patient we calculated the FC distance between its FC matrix at each measurement stage (2 weeks, 3 months and 1 year after the stroke incident) and the average FC matrix of the healthy controls (Fig. 4a). There is no significant difference between acute stage (2 weeks) and intermediate stage (3 months) (*t*(47) = 0.48, *p* = 0.63), while there is a significant difference between intermediate stage (3 months) and remote stage (1 year) (*t*(47) = 38.23, *p* < 0.01). Therefore, the FC distance with respect to healthy controls is a functional metric that indicates a progression of recovery across time. As an alternative approach, we calculated the Frobenius distance between the co-classification matrices (see “Methods”), resulting in a similar pattern as presented before. The only significant difference observed was between the second time point (3 months) and the third time point (1 year) when comparing their corresponding distances with the healthy control (Supp. Fig. 4).

In addition to the previous metric, we analyzed the relation between functional and structural connectivity, which exploits the anatomical data (Fig. 4b). There is a significant difference between healthy controls and patients in acute stage (2 weeks) (*t*(76) = 61.11, *p* < 0.01). There is no significant difference between acute and intermediate stage (*t*(44) = − 0.16, *p* = 0.86), while there is a significant difference between intermediate and remote stage (*t*(48) = − 42.62, *p* < 0.01) revealing a more similar level between controls and stroke patients, after 1 year of the incident. It is important to clarify that the structural information belongs to the healthy control template, and not to each individual patient.

The inclusion of the last two metrics (FC distance and FC/SC coupling) provides a comparison with traditional techniques in the field as reported in previous studies^59,60^. Therefore, it is important to incorporate such metrics in future studies in order to be able to compare the obtained results with previous literature.

#### Behavior

We inspected the recovery of the patients by using 3 behavioral recovery metrics (Fig. 4c).

We classified the recovery level of stroke patients between the ones with a higher (better) recovery against the ones with a lower (worse) recovery, by means of behavior as a division criterion (see “Methods” section). Reversibility in the latent space showed the highest accuracy performance (mean = 76%, SD = 09%), followed by mean FC in the latent space (mean = 65%, SD = 10%), reversibility in the source space (mean = 54%, SD = 12%) and mean FC in the source space (mean = 52%, SD = 13%). As an alternative approach, we used the first principal component of the recovery scores (see “Methods”) revealing a similar result. The highest accuracy performance was in the reversibility in the latent space (mean = 79%), followed by mean FC in latent space (mean = 77%), reversibility in the source space (mean = 58%) and last, FC mean in the source space (mean = 52%). These results are displayed in Supp. Fig. 6.

#### FC distance

The FC distance was used to divide the stroke patients with higher against lower recovery level. Reversibility in the latent space showed the highest accuracy performance (mean = 71%, SD = 11%), followed by mean FC in the latent space (mean = 64%, SD = 14%), reversibility in the source space (mean = 56%, SD = 12%) and mean FC in the source space (mean = 55%, SD = 12%).

#### Correlation FC SC

When classifying between higher and lower recovery level of stroke patients using the correlation between SC and FC as a division criteria, reversibility in the latent space showed the highest accuracy performance (mean = 70%, SD = 12%), followed by mean FC in the latent space (mean = 65%, SD = 12%), reversibility in the source space (mean = 58%, SD = 13%) and mean FC in the source space (mean = 55%, SD = 15%).

## Discussion

Deep learning models are being increasingly used in precision medicine thanks to their ability to provide accurate predictions of clinical outcomes from large-scale datasets of patients’ records. However, in the case of brain disorders, the deep learning approach is still limited, since clinical neuroimaging datasets typically have a small sample size. Thus, data scarcity has forced the adoption of simpler feature extraction methods, which are less prone to overfitting.

In the current study we tested whether by reducing the dimensionality of fMRI timeseries of stroke patients we retain clinically important features of the data. The analysis revealed that the functional features, such as modularity, that characterize the alterations caused by stroke, are preserved in the latent representation. Furthermore, the latent information proved to be efficient for clinical classification, discriminating between patients and controls, and between patients with low and high lesion volume at the acute stage. Moreover, the information of the latent space enhanced prediction of behavioral deficit at the acute stage and recovery after 1 year. These results demonstrate the clinical relevance of dimensionality reduction for brain disease and strengthen the case of its wider adoption to improve non only diagnosis, but also prognosis, hence allowing for a more effective treatment planning.

The results observed in Fig. 4 demonstrate the relevance of this approach for the patients’ treatment assessment. The prediction of recovery, when using the latent information (compared to the obtained using the source space) got enhanced. Therefore, the result informs on how embedded dynamics could be leveraged in order to get a more accurate baseline on the effects of the disorder at the moment of following the progression across time.

Our study contributes to the literature on dimensionality reduction approaches in neuroscience^61^. Reducing the dimensionality of the neural data is possible because different areas of the brain do not activate independently, but tend to fluctuate in coordinated patterns that can be described in terms of a smaller number of features^62^. However, a wide variety of dimensionality reduction methods are possible, and it is important to understand the relative strengths and weaknesses of the different approaches.

The most widely used approach is certainly PCA. However, PCA assumes linearity^63^. A way to tackle the limitations of the PCA (mainly the linearity assumptions), is employing deep learning techniques. These approaches have been increasingly used as a generic family of machine learning tools to learn features from fMRI data (see^64^ for a review). However, deep learning approaches are most effective in a supervised learning setting. In an unsupervised setting, autoencoders are more appropriate. At a theoretical level, our autoencoder has benefits over classical lineal procedures such as PCA. Autoencoders are non-linear and can learn more complicated relations between visible and hidden units^65^. A recent study used autoencoders to show that different brain states can be captures in terms of trajectories within a low-dimensional latent space^30^. In this study, the authors used a variational autoencoder, rather than a normal autoencoder, due to the necessity to represent new data not used in the training stage. In contrast, the current study does not need to include the variational feature to the autoencoder, as we encoded real data and analyzed what is obtained in the lower dimensions. By doing so, not only does the computational cost gets reduced but also the reconstruction becomes less complicated.

In our study, we find that the latent space time series retain several important properties of the original fMRI data, such as having common frames with high-amplitude co-fluctuations, as suggested in previous literature given the modular nature of the original FC^66^. And critically, the latent space data can be used to successfully classify stroke patients and stroke severity. These points underscore the idea that dimension reduction using the autoencoder framework is helpful, as it reduces the amount of data under investigation while simultaneously retaining relevant characteristics of the original data. Thus, the latent representation can be derived from the source space and by sharing the same embedded characteristics while discarding irrelevant information, improve the achieved classification accuracy.

One future direction is to explore how autoencoder-based dimension reduction can be employed in conjunction with whole-brain models and enhanced by using larger datasets (or simulated data). The mechanisms underlying the emergence of different brain states can be probed using whole-brain models based on conceptually simple local dynamical rules coupled according to empirical measurements of anatomical connectivity, for instance, by coupling nonlinear oscillators with the long-range white matter tracts inferred from diffusion tensor imaging (DTI)^30^. Previous studies have already demonstrated the utility of whole-brain models in stroke research^23,67,68^. The output of these models could similarly be embedded in a low-dimensional space, which could be analyzed using similar procedures to the ones described in this project.

Several previous studies have used dimensionality reduction to address stroke. A few studies used Artificial Intelligence to predict stroke incidents^69–71^ relying on linear procedures such as PCA for dimensionality reduction. Another study used dimensionality reduction approaches to associate motor and cognitive functions with mood disorders subsequent post-stroke^72^. In the study, the authors proposed a non-linear model that effectively predicted post-stroke neuropsychiatric symptoms, outperforming traditional linear classifications. Another study proved that distinction between patients with post-stroke vascular dementia and control subjects was enhanced by using a dimensionality reduction technique^73^. Furthermore, a recent article showed how four well-known dimensionality reduction techniques can be used to extract relevant features from resting state functional connectivity matrices of stroke patients^74^. Nevertheless, their proposed approach relies on linear assumptions in contrast to the ones selected for our study.

To date, machine learning has not been applied to explore in depth the low dimensionality of stroke effects in the brain. One study implemented an unsupervised features learning approach based on an autoencoder for automatically segmenting brain MR images from stroke lesions^75^. Nevertheless, the study focused only on anatomical information without considering functional data. Our study is thus novel in that it tries to achieve a low-dimensional representation of the functional data^14,76–78^. While autoencoders are routinely used for dimensionality reduction across a wide range of fields, their usage in functional neuroimaging is still scant. Our findings demonstrate the large, and still underexploited potential of machine learning methods in the study of large-scale brain dynamics. Our novel approach may be fruitfully applied to a wide array of brain disorders, subserving both the theoretical goal of a clearer understanding of these diseases, and at the same time, the clinical goal of maximizing patients’ classification, diagnosis, and prognosis.

In the present study, we reached high classification accuracies by only relying on functional data. In addition to the classification of low dimensional description of functional data, not only fMRI, but also EEG, could be quite helpful to select target for non-invasive brain stimulation. Currently, there have been proposals to use patterns of functional connectivity^79^ to guide invasive and non-invasive brain stimulation. However, high dimensional data sets like resting-state fMRI connectivity patterns are difficult to collapse in a small set of coordinates. The low dimensional embedding coupled with classification methods to highlight the most predictive components could be a strategy to select sensitive targets.

It is important to note that nonlinear dimensionality reduction methods are often fragile in the presence of noise^3^ in the presence of low data quality, which limits their use when statistics is limited. However, datasets with sufficient length, such as the one presented in this article, can avoid this problem. Therefore, before proceeding to nonlinear methods, it is worthwhile to ensure a dense enough sampling of the high-dimensional space such that local neighborhoods include data points from different trajectories.

In our current study, we used an autoencoder model because of its ease-of-use and flexibility. However, there are other interpretable variants that have been proposed to enable the inspection of embedded information^80,81^. These methods incorporate additional priors to encourage separability across latent dimensions. As our approach is relatively general, exploring deep representations could provide a way to visualize the representations formed in generative models for other applications in medical imaging. With new strategies for interpreting how deep networks represent data, we may be able to develop new regularization strategies to disentangle and interpret population-level variability. Moreover, future studies could design visualization techniques in order to interpret the features extracted by non-linear dimensionality reduction, which could provide valuable insights to the clinicians for the design of more effective rehabilitation protocols.

We selected a whole-brain metric, the average of the FC, in order to perform the classification and prediction analyses. Even though this metric is outperformed in literature by other stroke-related metrics^13^, we used it in order to be comparable to the performance in the latent representation, as when data gets reduced, the anatomical properties such as space localization, get lost.

Lastly, whole-brain models introducing anatomical information^24^ could be applied directly to the latent representation in order to add anatomical restrictions and enrich the information available for the proposed analysis.

In conclusion, this study adds new evidence for the relevance of low-dimensional embeddings for fMRI signals and proposes a non-linear dimensionality reduction approach as a promising tool to explore altered brain dynamics after stroke. In addition, it reveals how complexity metrics such as brain signal reversibility provide indicators that relate to lesion severity and predict lesion recovery, making it the first study of this type with applications to longitudinal stroke studies. Our findings demonstrate the power of a measure of brain signal reversibility as a general marker of pathology. While many complexity measures have been used as biomarkers in functional MRI, our measure has the twofold advantage of its simplicity and interpretability. In fact, the degree of signal reversibility is a general measure of how distant a physical system is from thermodynamic equilibrium. Healthy cognition is associated with complex information processing demanding a far-from-equilibrium state^82,83^. Therefore, a higher reversibility holds as a strong marker of neurophysiological impairment. We expect similar results in a wide array of neurological and psychiatric conditions.

### Supplementary Information

Supplementary Information.

## Data Availability

Data is available upon request. Code used for the analysis are available at https://github.com/SebastianIdesis/Latent_Space_Stroke-2023-.
