# Supplementary material for: A low dimensional embedding of brain dynamics enhances diagnostic accuracy and behavioral prediction in stroke
Source: Sci Rep. 2023 Sep 21;13:15698. doi: 10.1038/s41598-023-42533-z (PMC10514061; doi:10.1038/s41598-023-42533-z)
Supplement: Supplementary file 1 — Supplementary Information. [file 41598_2023_42533_MOESM1_ESM.docx]

**SUPPLEMENTARY FIGURES**

*
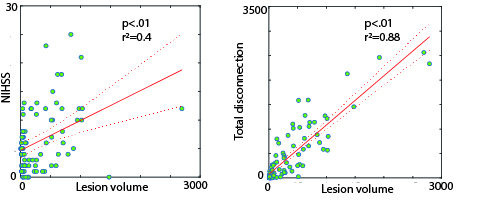
*

*Supplementary Figure 1*: Correlation between Lesion volume and NIHSS (Left) and Total number of disconnected tracts (Right). Both relations showed a significant relation probing the robustness of the elected metric.


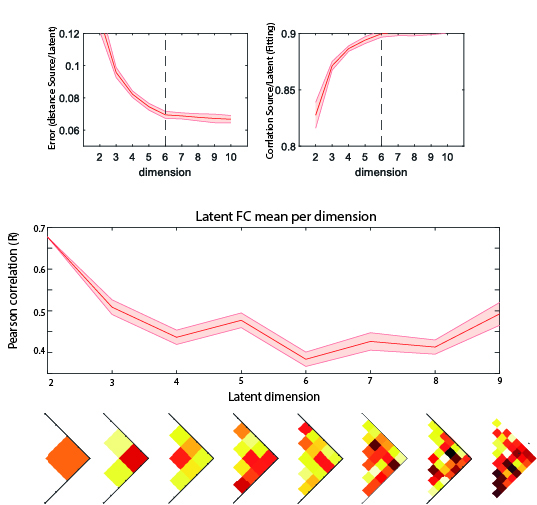


*Supplementary Figure 2*: Reconstruction error and Latent FC per dimension: (A) Reconstruction error reached a level of 0.06 at dimension 6 showing no rate of change higher than 0.005 after it. Fitting level reached a level of 0.89 showing no rate of change higher than 0.005 after it. (B) The highest correlation between the latent FC and the source FC is shown at dimension 2 (r= .67) while the lowest correlation value is shown at dimension 6 (r=.38)


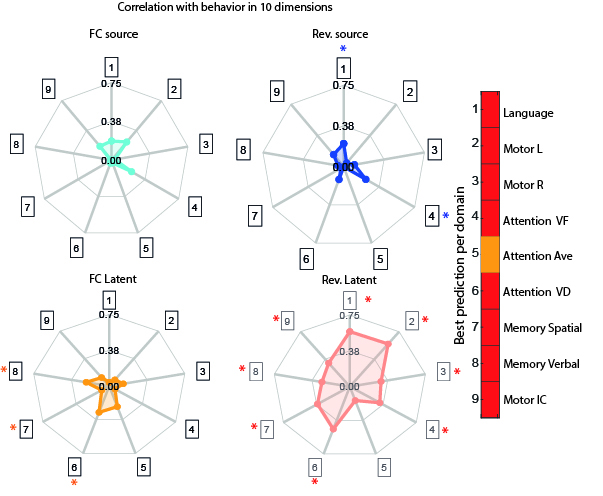


*Supplementary Figure 3*: Association between dimension 10 and behavioral scores. For each behavioral domain, the corresponding metric with the highest association was represented indicating the respective color. Red represents the latent space metric while blue represents the source space.


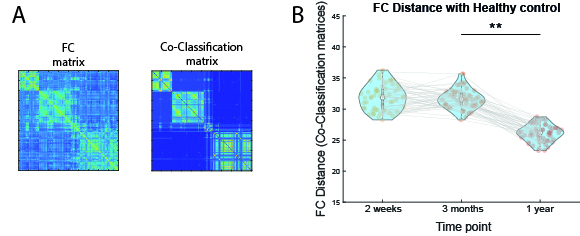


*Supplementary Figure 4*: Co-classification matrix reveals same pattern as empirical FC when compared with healthy controls across time. This analysis was performed in comparison to the one presented in **Figure 4a.**


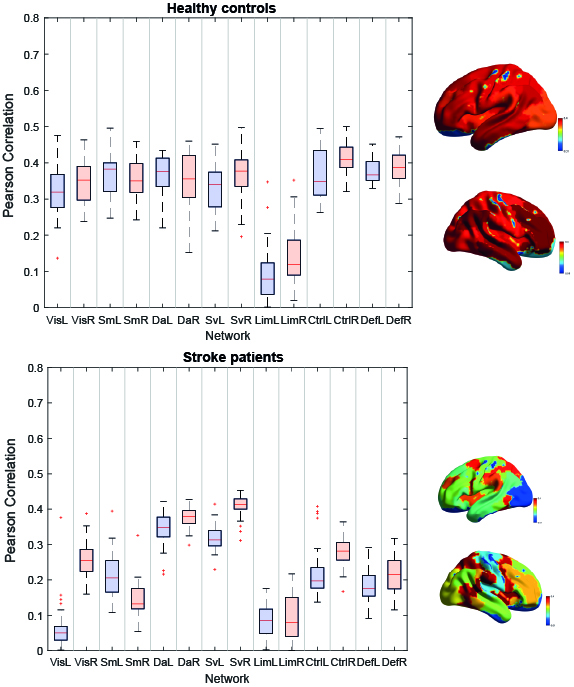


*Supplementary Figure 5*: SC-FC correlation per network: The relation between structural and functional information of networks reveals a symmetry between homotopic regions in the healthy control and asymmetry in the stroke patients’ group


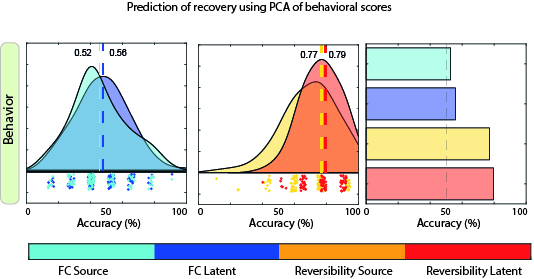


*Supplementary Figure 6*: Prediction of recovery using the first principal component as separation criteria.


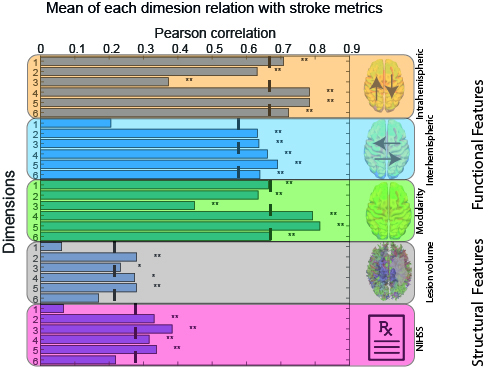


*Supplementary Figure 7*: Association between latent representation pattern and structural/functional features: We calculated the Pearson correlation R value for each dimension mean and standard deviation, with the corresponding metric of interest. By studying the mean of each of the 6 of the latent dimensions, intrahemispheric value showed a significant relation with all of them, interhemispheric with 5 of them, modularity with all of them, lesion volume with 4 of them and NIHSS with 4 of them.

*
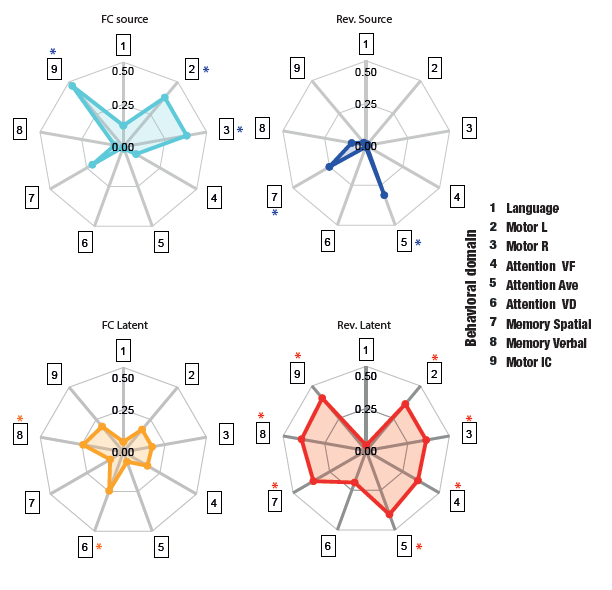
*

*Supplementary Figure 8*: Correlation of mean FC with behavior impairment at acute stage (2 months after stroke incident): Each behavioral domain at the acute stage was related with the mean FC and reversibility in both source and latent space. Asterisks indicate the significant relations. The reported values are as follows: FC Source: .12, .38, .38, .08, . 01, .001, .21, .05,.46; Rev Source: .003, .01, .01, .002, .31, .008, .25, .08, .02; FC Latent: .05, .17, .17, .16, .06, .24, .09, .24, .19; Rev Latent: .03, .36, .36, .35, .40, .20, .36, .28, .40.

*
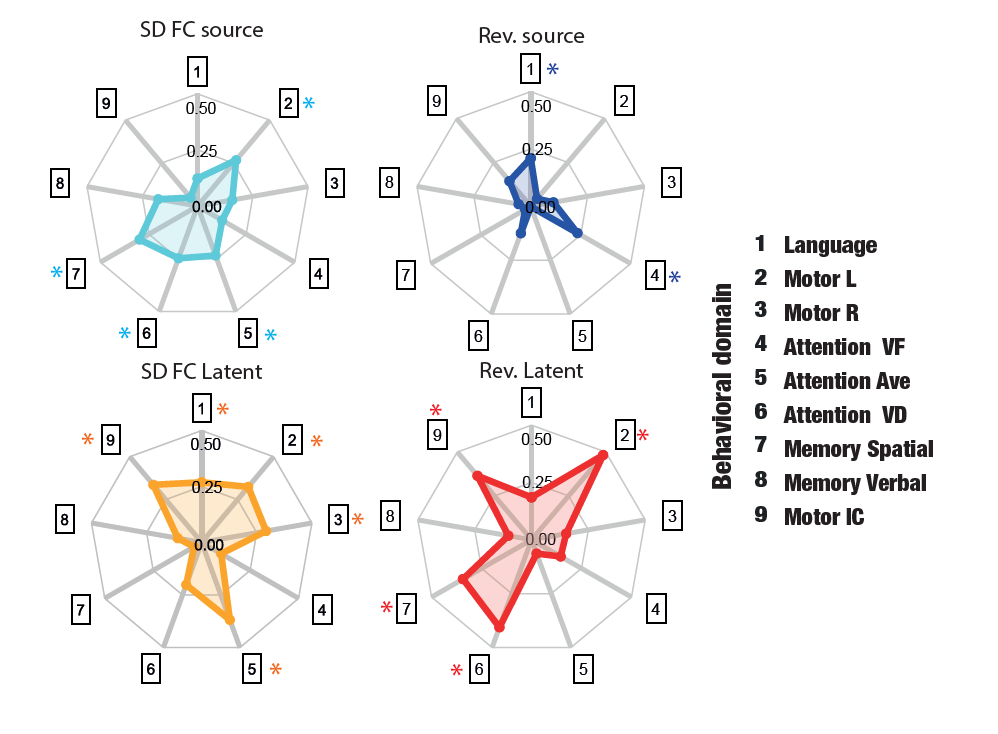
*

*Supplementary Figure 9*: Correlation of FCs’ standard deviation with behavior impairment recovery: All the metrics used for the classification approach were related with each of the 9 behavioral domains recovery values (score after 1 year minus score after 2 weeks, divided the 2 weeks score). Asterisks represent which of the relations were significant. The reported values are as follows: FC Source: .24, .27, .16, .29, . 07, .33, .24, .15,.22; Rev Source: .04, .001, .01, .05, .00, .01, .00, .00, .02; FC Latent: .02, .32, .29, .10, .36, .20, .03, .10, .33; Rev Latent: .18, .06, .28, .21, .07, .14, .23, .07, .26.

*
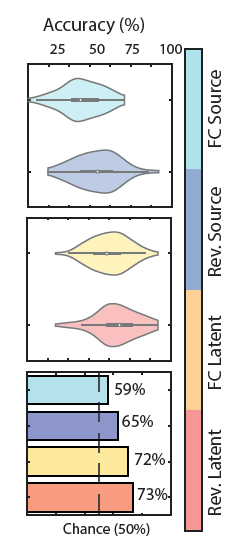
*

*Supplementary Figure 10*: Classification of severity using NIHSS as division criteria: The distinction between stroke patients with low and high lesion volume indicated that the highest accuracy was given by the reversibility in the latent space (mean = 74%).


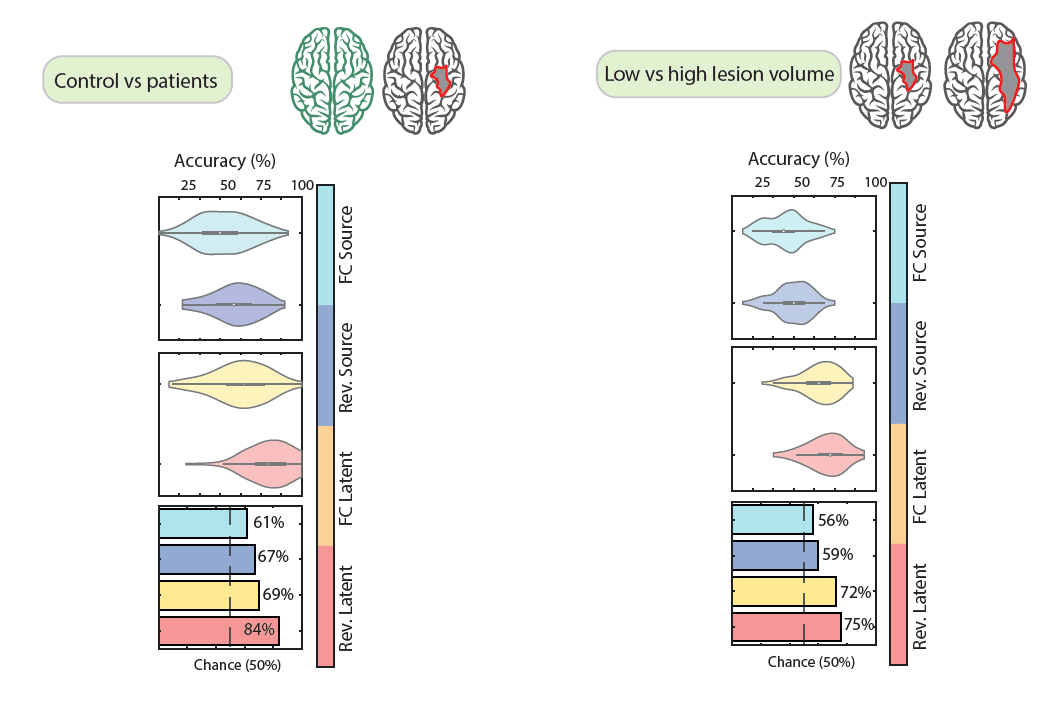


*Supplementary Figure 11*: Classification using the standard deviation instead of the FC average: The distinction between stroke patients with low and high lesion volume and between healthy controls and patients showed the highest accuracy in both cases in the reversibility of the latent space.

*
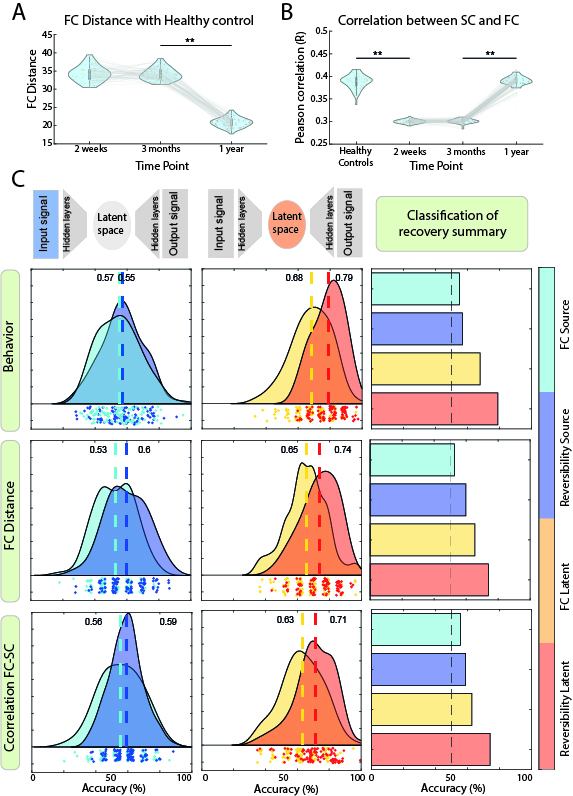
*

*Supplementary Figure 12*: Prediction of recovery using the standard deviation instead of the FC average: **(A)** FC distance (Frobenius norm of the difference between the two matrices) between stroke patients at each time point and the healthy controls indicating the decrease of distance after 1 year (p<.01). **(B)** Correlation between SC and FC of healthy controls and stroke patients (at each measurement stage) revealing the increase at the remote stage, showing a similar value to controls after 1 year of the incident (p<.01)., while it is not the case after 2 weeks and 3 months (p=.86). **(C)** Prediction of recovery using as input of the classifier the FC’s standard deviation and reversibility matrix of the source space and the FC and reversibility matrix of the latent space. To split the subjects in high vs low recovered, 3 different criteria were used: Amount of behavioral domains recovered, the FC distance at remote stage and the correlation between SC-FC at remote stage. In all the scenarios, reversibility in the latent space showed the highest accuracy.


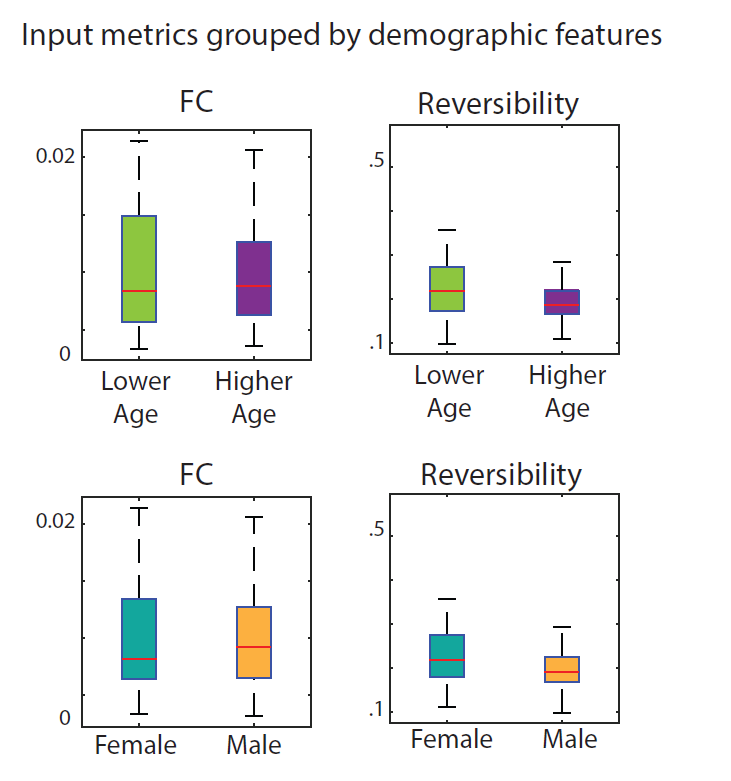


*Supplementary Figure 13*: Input metrics grouped by demographic features: In order to assess the influence of demographical factors, we calculated the classifier input metrics (FC and reversibility) for subgroups divided by the median age (top subpanels) and by gender (low subpanels) showing no significant differences (*p*>.2) between the groups.


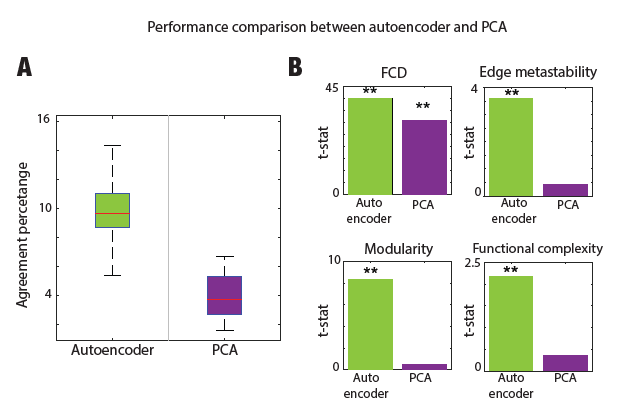


*Supplementary Figure 14: Preserved features in latent space compared to PCA performance: (A) Agreement percentage was compared for the 6 latent dimensions and the 6 first principal components, showing a higher performance of the autoencoder. (B) Difference between the healthy control and the stroke patients was assessed in the latent space and in the 6 first principal components for all the studied metrics. The difference in metrics was significant for each application of the autoencoder while the only significant difference in metrics for the application of PCA was for FCD.*

Table 1

|  | Healthy Control | Stroke patients |
| --- | --- | --- |
| Age | 56.6 | 52.3 |
| Gender | M=59% F=41% | M= 57% F=43% |
| Education | 13.9 | 13.4 |
| Handedness | R=91% L=9% | R=94% L=6% |

*Table 1:* Demographic information of the involved subjects. For more information and detailed scores in behavioral tasks, see (Corbetta et al., 2015)
